# Three Giants in the Cradle of Reproductive Medicine; Reproduction Theories of the Seventeenth Century as Discerned by Pregnancy Portraiture in the Oeuvre of Jan Vermeer

**DOI:** 10.5041/RMMJ.10244

**Published:** 2016-04-19

**Authors:** Ronit Haimov-Kochman, Irving M. Spitz

**Affiliations:** 1 Department of Obstetrics and Gynecology, Hadassah Hebrew University Medical Center, Jerusalem, Israel; 2 Institute of Hormone Research, Shaare-Zedek Medical Center, Jerusalem, Israel

**Keywords:** Art, history, medicine, pregnancy, reproduction

## Abstract

Portraits of pregnant women are rare in Catholic Renaissance art. In seventeenth-century Holland, the Catholic rule of Spain had been thrown off and a Protestant Calvinistic republic emerged, freeing Dutch artists to choose an unorthodox subject matter for their paintings. The Golden Age of Holland was characterized by extreme wealth, originating from overseas trade, which fostered a marked interest in philosophy, science, medicine, as well as art. Despite this, portraiture of pregnancy remained uncommon. An exception to this rule was Jan Vermeer of Delft, who lived during the zenith of this era. Jan Vermeer painted fewer than 40 pictures, fathered 15 children, and died bankrupt and little appreciated at the age of 43. Vermeer confined himself almost entirely to images of women in various domestic situations, including three figures of pregnant women. In this framework, pregnancy could be viewed as an icon for fidelity and conformism to social expectations. In this paper we investigate the roots of this unusual icon in Vermeer’s oeuvre, and suggest that the use of pregnancy in his paintings could have been inspired by his Delft-resident contemporaries Antony van Leeuwenhoek and Reinier de Graaf, fathers of well-known and opposing theories of reproduction. These eminent scientists and Vermeer’s pregnant wife, who frequently served as his model, might have made pregnancy less mysterious and more realistic to the painter.

## INTRODUCTION

Portraits of pregnant women are rare in Italian Renaissance paintings due to the influence of Catholicism. However, in Holland in the seventeenth century, the Catholic rule of Spain had been thrown off, and a Protestant Calvinistic republic emerged. The stern Calvinistic creed had little use for religious art; therefore, Dutch artists had the freedom to choose themes for their paintings.^1^ This Golden Age of Holland, which spanned most of the seventeenth century, was characterized by sharp realism that permeated philosophy, science, medicine, and art. The United Provinces of Holland, where the quest for knowledge was not seen in conflict with religion, became one of Europe’s foremost scientific centers. It was in Leiden that René Descartes had published his famous *Discourse de la Méthode*. The first astronomical observatory was erected in the University of Leiden at the same time as, in Italy, Galileo was standing trial for his views. In Amsterdam, Dr Nicolaes Tulp was depicted by Rembrandt during an anatomy lesson, dissecting the forearm of a cadaver in front of an anxious audience (*The Anatomy Lecture of Dr. Nicolaes Tulp*, c. 1632, Mauritshuis, The Hague). Such a scene could have taken place only during the Age of Reason within a society highly appreciative of medical investigation.^1^

Thanks to overseas trading, Holland was elevated to one of Europe’s great powers and experienced tremendous economic, social, and political growth. Indeed, by the middle of the seventeenth century, half of Europe’s trade was carried by Dutch ships. The general tenor of life was set by the merchants who experienced great economical satisfaction, as mirrored by their self-portraits. The spectacular rise in fortune provided a livelihood for a multitude of artists. Turning away from the religious, mythological, and allegorical themes of Renaissance art, and having a vibrant market to sell their production, they portrayed their surroundings, celebrating everyday life with unblinking directness.^1^,^2^

Thus, it is intriguing to find so little illustrative evidence of pregnant women in Flemish-Dutch portraiture at a time when contraceptives were non-existent, and multiparity was the rule. Moreover, sometimes advanced pregnancy was even concealed, as in the case of Reynu Meynertsdr Semeyns, who was portrayed by Jan Claesz on the occasion of her marriage and gave birth four and a half months later. However, her pregnant state was not indicated in the portrait.^3^,^4^ Pregnancy was sometimes addressed as unforeseen in theatrical commedia dell’arte scenes of young lovesick women seeking medical advice from quack doctors (Jan Steen, *Love Sickness*, c. 1660, Alte Pinakothek, Munich; *Doctor’s Visit*, c. 1663, Apsley House, London). The diagnosis of early pregnancy was made by visual examination of the patient’s bottled urine, and, rarely, by “reading” the smoke coming from burning coals in the patient’s basin. Midwifery manuals list a large number of physical signs of pregnancy—none of which were, however, certain. A lively birth celebration is another amusing setting that served as a decoration of pregnancy (Jan Steen, *Celebrating the Birth*, c. 1664, Wallace Collection, London). These women’s gatherings, full of loquaciousness, laughter, and drinking, were disreputable for transgressive talk led by midwives, who moved among households and were purveyors of bawdy gossip.^3^ It seems that the still mysterious nature of human reproduction intimidated both the artists and their audience, and led to either denial or humorous treatment of pregnancy.

**Figure 1:**
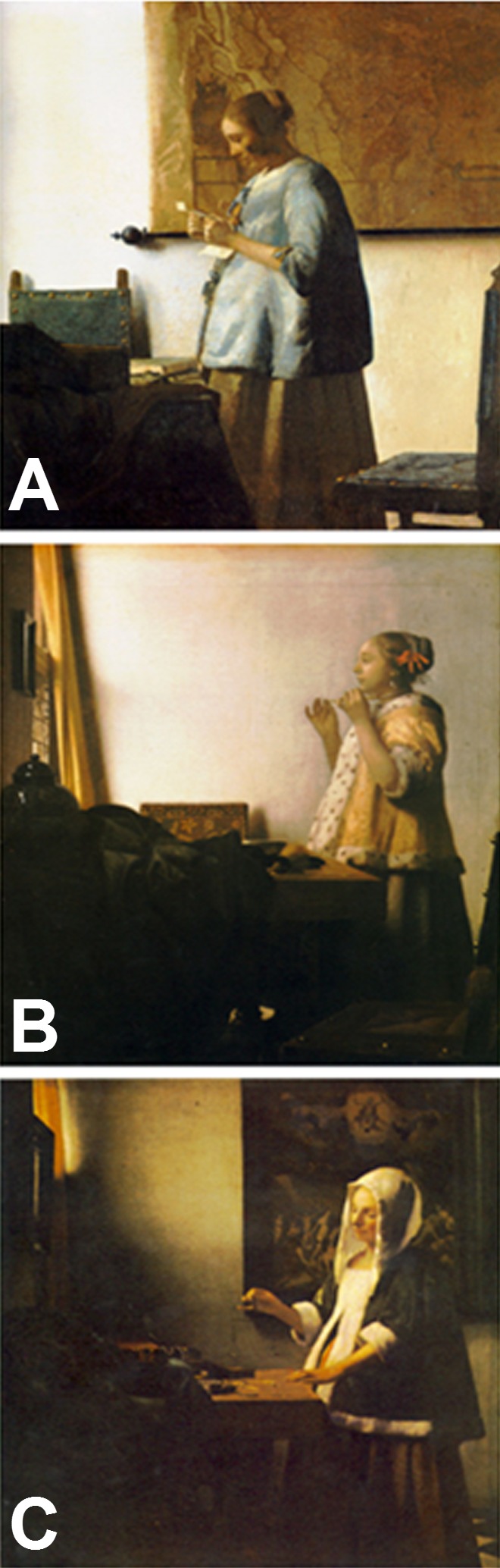
Jan Vermeer. A: *Woman in Blue Reading a Letter*, c. 1662–1664, Rijksmuseum, Amsterdam. Public domain via Wikimedia Commons. B: *Woman with a Pearl Necklace*, c. 1664, Staatliche Museen zu Berlin. Public domain via Wikimedia Commons. C: *Woman Holding a Balance*, c. 1662–1664, National Gallery of Art, Washington DC. Public domain via Wikimedia Commons.

Jan van der Meer (Joannes Vermeer), in marked contrast to his contemporaries, depicted gravid women in a realistic manner (Figure 1). He was born in Delft in 1632, near the zenith of the Golden Age, and died in 1675 at the age of 43, when this era was coming to its end. Vermeer was recognized as an artist by the St Luke guild by the age of 21. He married Catharina Bolnes and fathered with her 15 children, of whom 4 died when still very young. Producing on average only two paintings a year (fewer than 40 paintings are attributed to Vermeer today), he could scarcely have met the high cost of living from selling his works. For further financial support for his household, Vermeer worked as an art dealer and as an innkeeper. His later years were overshadowed by a dramatic deterioration of his financial position. He died apparently little appreciated or esteemed; it was 200 years before his luminous paintings were recognized as great masterpieces of Western art.^1^,^2^,^5^ This paper investigates the possible inspiration of personal experiences and acquaintances with leading figures of human reproductive history that might have brought the theme of pregnancy to the attention of Jan Vermeer and made it less mysterious and more approachable to him.

## PREGNANCY PERSPECTIVE AT THE GOLDEN AGE

Medical illustrations from the seventeenth century still depicted the pregnant stomach as a bud of a blossom opening up, petal for petal.^6^ The similarity of human gestation to a seed giving fruit was portrayed by Leonardo De Vinci in his famous anatomy study on the gravid womb.^7^ During the seventeenth century, long-established views on human conception were uprooted by newly made discoveries. Surprisingly, the fathers of these discoveries and evolving novel concepts were immediate neighbors of Vermeer in Delft, Antony van Leeuwenhoek and Reinier de Graaf.

### Antony van Leeuwenhoek and the Discovery of the Spermatozoon

**Figure 2:**
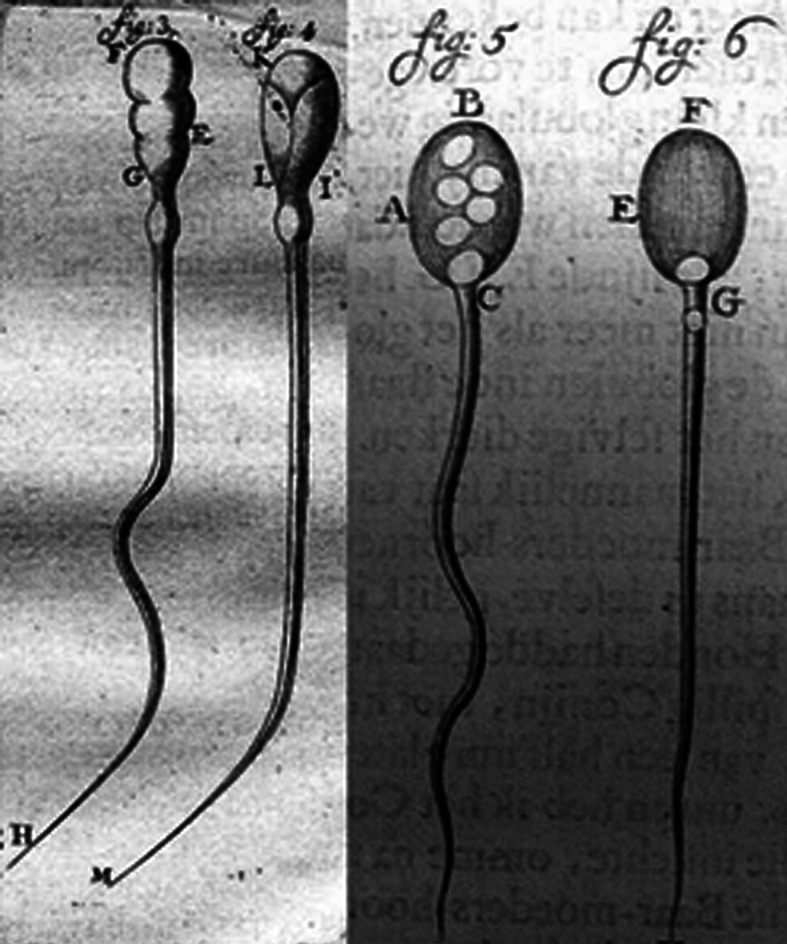
Antony van Leeuwenhoek. Human and rabbit spermatozoa, 1678, from a letter to the Royal Academy of Sciences. From Wellcome Library London; Wellcome Images. Reproduced under Creative Commons Attribution license CC BY 4.0.

**Figure 3:**
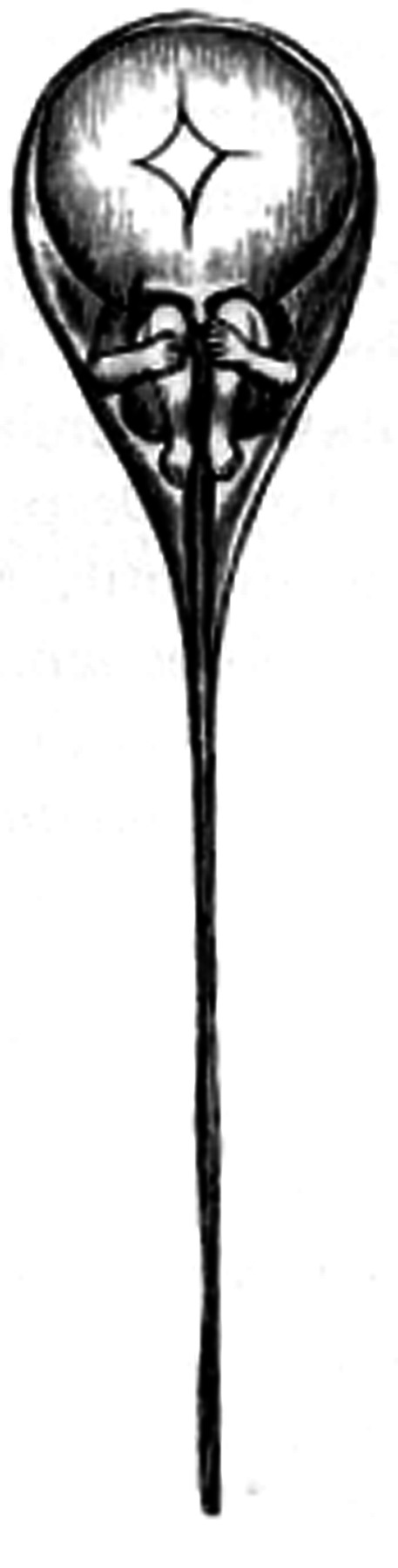
Nicolaas Hartsoeker. Sketch of a homunculus enclosed in a human spermatozoon, 1694, from *Essai de Dioptrique*, Paris. Public domain via Wikimedia Commons.

Antony van Leeuwenhoek (1632–1723) was a tradesman of Delft, who developed about 400 microscopes, some with a magnification power of ×270. His studies on microscopy led him to the first description of male spermatozoon. In 1677, Johan Ham, a student of the medical school in Leiden, handed van Leeuwenhoek a semen sample from a patient inflicted with gonorrhea. Both Ham and van Leeuwenhoek observed the *lǘtgen dierkens* (tiny creatures) as a component of male sperm. Van Leeuwenhoek resumed his observations in his own semen, examined immediately following conjugal coitus, describing a multitude of “animalcules,” less than a millionth the size of a grain of sand and with thin undulating transparent tails.^8^ In his letters to the Royal Society in London he included drawings of the spermatozoa (Figure 2) that were the first known drawings of male sperm cells.^6^ Van Leeuwenhoek rejected the idea of *de novo* creation of organisms, out of thin air (*Generatio Spontane* of Aristotle, 384–322 BC). In his writings describing conception, procreation, and development of the human embryo, he speculated that these cells hold the seed of human life. He claimed that the entire form of the fetus (*Homunculus*) was enclosed in the spermatozoon (Figure 3), but also confessed that he was unable to prove this.^7^ He was hoping to be fortunate enough to find an animal whose “male seed” was large enough to allow recognition of the figure of the creature within it. Later, at the turn of the century, he speculated further that the miniature replica in the spermatozoon was assembled only when nourished in the womb. The female uterus was viewed then as nothing but an incubator, which protected and supported the fetus during gestation. Van Leeuwenhoek’s ideas were in accordance with the views of Aristotle who separates male and female roles in reproduction into active and passive, respectively. He believed that the female furnishes the material for the embryo which is made of the female menstrual blood, defining the material cause of generation.

The efficient cause is defined as what causes existence, and for reproduction Aristotle designates the male semen as the efficient cause. The male is the one who passes on the principle of life or the soul (*psyche*), the generative agent, whereas the female provides only the food for the developing embryo.^6^

### The “Ovist” Theory of Reinier de Graaf

Reinier de Graaf (1641–1673) was a respectable physician in Delft at that time. In 1672, he published his pioneering work *De Muliebrum Organis* on female anatomy. The prevailing thinking in the Netherlands at that time was that the female “semen” derived from the female “testicles” and reached the uterus via the fallopian tubes. In 1667 observations of de Graaf led a circle of anatomists affiliated with the medical school of Leiden, including Jan Swammerdam and Dane Nicolaus Steno, to redefine the female testicles as ovaries and the ovarian follicles as eggs.^8^ Shortly thereafter, it was de Graaf who first recognized that the true egg was in fact much smaller than the whole follicle and that it was capable of passing through the fallopian tubes during its migration to the uterus. His study argued against the old procreation theorists and proposed a new theory of human reproduction, known as the “*ovist*” theory. De Graaf assumed that male spermatozoa contain the spirit of life which was infused into the ovum. He did not foresee that human life begins with fusion of egg and sperm. However, the discovery of the ovarian follicle (now named the Graafian follicle), the ovum, and their role in conception changed the concept of fertilization to recognize that a woman also participates in the creation of her offspring. This theory was very provocative at that time and had not prevailed since the time of Hippocrates two thousand years earlier. Hippocrates (460–377 BC) postulated that a seminal fluid that is formed both in men and women flows into their sex organs. Following copulation, the two fluids mix and create new life. During the Middle Ages, the female was predominantly described in terms that emphasized her receptive, protective, and nourishing functions. Her body served as a *broedruimte* (incubator) for the fetus; her uterus was but a *vas* (vessel) or a *cavitas.* The postulation that the spirit of life was contained in the spermatozoon was based on an argument dated from the Baroque period that every form of life expresses itself through movement. The *ovist* theory could be discarded by a quick look through the microscope: the liveliness of sperm compared to the stationary egg.

The lives of Antony van Leeuwenhoek, Reinier de Graaf, and Jan Vermeer appear to intermingle. Following the untimely death of Vermeer, Antony van Leeuwenhoek was appointed as curator to handle the bankruptcy declared by Vermeer’s widow. Both van Leeuwenhoek and Vermeer were born in Delft in 1632 in the same week, and both are documented on the same page of the municipal records of the city of Delft. Delft was then the fourth most populous town in Holland with 22,769 inhabitants (by the population count of 1622).^9^ More importantly, the father of Vermeer was a textile merchant in Delft, as were van Leeuwenhoek and his father-in-law. It is also suggested that van Leeuwenhoek was a model for Vermeer’s paintings *The Geographer* and *The Astronomer*, his only paintings with predominant male figures.^2^,^6^ Both van Leeuwenhoek and Vermeer had a passion for optics and for the properties of light. Vermeer was believed to have used a *camera obscura* for bringing details into his paintings following a suggestion by van Leeuwenhoek. Both van Leeuwenhoek and Vermeer had captured light and sculptured it; the former conducted it through the microscope lenses in order to magnify an image without losing its sharpness, and the latter accentuated light in order to achieve depth in his paintings without sacrificing the brightness of the colors. According to C.C. Dobell, a biographer of van Leeuwenhoek, the drawings of the spermatozoa (Figure 2) that were sent to the Royal Society in London were in fact made by Vermeer.^6^ As a member of the Royal Society in London, Reinier de Graaf was the one who introduced Antony van Leeuwenhoek to this distinguished circle. Both men attended the anatomic lessons held every week at the Delft Anatomy Theatre, which shared its locale with Delft’s civic guard, of which Vermeer was an active associate member.^6^ The major dispute between the two investigators about the origin of life that took place presumably at Vermeer’s own inn in Delft could have inspired Vermeer. Therefore, pregnancy as a major theme in Vermeer’s art might have been less mysterious and more approachable to him.

### Vermeer’s Attitude towards Women and Pregnancy

The artistic treatment of women is pivotal to Vermeer’s work. During his major period of productivity, he abandoned most extraneous subjects and confined himself almost entirely to the image of the young woman, alone, in various domestic situations. About 30 out of Vermeer’s works depict women as the predominant figure. Hasty observers come away from a perusal of his paintings with the impression of the “sacredness of the woman, who creates the happy and well-ordered home.”^1^ Alternatively, one can easily view Vermeer’s figures as trapped in domestic captivity. Vermeer, the alleged poet of domesticity, had never failed to fill his paintings with moral messages.^5^ Symbolic icons were a common means for conveying a non-verbal message at that time. The women in his paintings either practice humility and modest introspection, conforming to the official code of thought and behavior of the *exemplum virtutis* (model of virtue), or violate the norms by yearning for extramarital relations, which break the vow of chastity. The iconography of the moral conflict could be reflected in wine as a love potion, fruits and music of seduction, extramarital love letters, jewelry of vanity, open passages that let in the evil temptation of the soul, or in objects such as the pure white milk, and the water and bread of life of moderation and simplicity.^2^,^5^ A woman subjected to such a conflict is depicted in *Woman in Blue Reading a Letter* (Figure 1A). A pregnant woman faces the light pouring in from an invisible window, completely engrossed in reading a letter, her cheeks moistened. The intruding light and love letters were a frequent theme in Vermeer’s work, pointing to adultery. Her advanced gravid state stands for the respectability of marriage, as an institution designed to ensure reproduction. Such a pronounced pregnancy brings about connotations of fidelity, fertility, dependency, and conformism to social expectations and the spouse’s demand of monogamy.

Another pregnant figure is seen in *Woman with a Pearl Necklace* (Figure 1B). She stands in solitude in front of a mirror, adorning herself with pearls. Pearls take on many symbolic meanings, ranging from purity, faith, and virginity, to the vices of greed and arrogance. In his *Introduction to the Devout Life* (1608), published in a Dutch translation in 1616, the mystic St Francis De Sales addressed spiritual meaning in the white, flawless luster of pearls.^5^ Pearls were also the attribute of St Margaret of Antioch, the holy patron of pregnancy and childbirth. Childbirth was hazardous in that period when one in a hundred women died as a direct result of labor. Part of the St Margaret of Antioch popular cult was the promise that women in childbirth upon calling on her would be safely delivered. The coronet she wears features pearls which are a symbol often shown with Margaret because her name in Greek means “pearl.” Self-adoration with pearls could be intended to attract St Margaret’s blessing during the hazards of pregnancy.^10^

While generally accepted as an allegory, *Woman Holding a Balance* (Figure 1C) has been interpreted in many ways over the years. In a darkened room a gravid woman is balancing empty scales; against this gloom the pearls in the boxes have a sparkling glitter. In view of the advanced stage of gestation, and following an old folk tradition, the act of weighing pearls was carried out in order to divine the sex of the child to be born or to judge the fate of his soul.^2^ The picture on the wall behind the figure is a Last Judgment. This background provides a theological context for the scales she holds: to judge is to weigh. According to Christian teaching, on the Day of Judgment, good and evil will be sundered. The apocalyptic scene is an eschatological appeal to the conscience of the woman, plainly bearing a semantic relation to her thoughts and actions. Although the scales are empty, the jewelry boxes, the pearls, and the gold are valued within the temporal world. As such, they represent temptations of material splendor.^5^ The vacant balance, the serenity and inner peace that the woman exudes, and her evident pregnancy provide an optimistic view to the picture. Indeed, this painting embodies pregnancy as an emblem of reproduction and continuity.

Since there are only a few examples of pregnant women in Flemish-Dutch portraiture, it was questioned repeatedly in the literature on Vermeer whether these women were actually pregnant. It was suggested that the protruding stomach was merely the result of fashion, as they were wearing a crinoline farthingale, a hoop skirt, as is proposed by the painting *Girl in a Blue Dress*, *c.* 1641, by Johannes Cornelisz Verspronck at Rijksmuseum, Amsterdam, which features a young girl wearing such a dress.^4^ The formal gown, *tabbaards*, was a combination of a stiffened bodice and a matching skirt and was impossible to wear during pregnancy. In fact in 1659 Jesuit Adriaen Poirters urged married women to avoid the use of such bodices as they could cause miscarriages.^3^ Most of the women depicted in Vermeer’s paintings are wearing informal, everyday clothes that may serve as maternity dresses.

To the professional eye of the obstetrician, the pregnancy is invariably evident. It was apparently clear to the artistic eye of Vincent Van Gogh, who vividly described *Woman in Blue Reading a Letter* (Figure 1A) in 1888 to his colleague Emile Bernard: “Do you know a painter named Jan van der Meer? He painted a pregnant Dutch woman, beautiful and very distinguished. The palette of this strange painter is blue, citron yellow, pearl gray, black and white”.^1^,^5^ Moreover, it is conceivable that the model for the pregnant women was Vermeer’s wife. Due to financial constraints and slow rate of productivity, Vermeer could not afford a professional model. She gave birth to 15 children during 20 years of marriage and was therefore pregnant most of the time.^1^

## THE CANVAS AS A *SPECULUM SINE MACULA*—ART AS A MIRROR OF CULTURE

Community standards and intellectual fermentation among peers can be major sources of influence, as are domestic conditions and quantity of artistic output. The free scholastic debate about human procreation and reproduction might have been the atmosphere in which Vermeer functioned. Pregnancy was no more virtual or metaphysical; it was at the center of aristocratic dispute led by two major figures in Vermeer’s hometown. In view of the moral conflicts depicted within Vermeer’s paintings, pregnancy stands as an icon that is intended to intensify the experience of ambivalence in marriage life. The canvas of Vermeer discloses the social attitudes towards women in general and pregnancy in particular. It may also unveil the academic dispute between the two pivotal scholars who profoundly contributed to the understanding of human reproduction during the Age of Reason in Holland of the seventeenth century.

## References

[1] Koningberger H (1967). The World of Vermeer.

[2] Vermeer Nash J (2002).

[3] De Winkel M, Gaskell I, Jonker M (1998). The Interpretation of Dress in Vermeer’s Painting. Vermeer Studies.

[4] De Jongh E (1986). Portretten van echt en trouw. Huwelijk en gezin in de Nederlandse kunst van de zeventiende eeuw. Cat. 15. [Exhibition catalogue Frans Hals museum].

[5] Schneider N (2000). Vermeer the Complete Paintings. 1632–1675. Veiled Emotions.

[6] Leonhard K (2002). Vermeer’s pregnant women. On human generation and pictorial representation. Art History.

[7] Garrard MD, Broude N, Garrard MD (1992). Leonardo da Vinci: Female Portraits, Female Nature. The Expanding Discourse: Feminism and Art History.

[8] Ruestow EG (1983). Leeuwenhoek’s perception of the spermatozoa. J Hist Biol.

[9] Montias JM (1979). Statistical evidence on the economic status of artists and artisans in Delft in the 17th century. Journal of Cultural Economics.

[10] Cork R (2003). Pearly queen. Tate Magazine.

